# A Case Report and Literature Review of Pyopericardium and Its Complications

**DOI:** 10.51894/001c.144439

**Published:** 2025-09-30

**Authors:** Anthony Cook

**Affiliations:** 1 Department of Internal Medicine Henry Ford Warren

## 73

### INTRODUCTION

Acute pericarditis is most frequently caused by viral infections, such as Coxsackie virus. Bacterial pericarditis, also termed pyopericardium, occurs in less than 1% of pericarditis cases. Though virtually unseen in the age of easily accessible antibiotics, pyopericardium cases reach mortality rates of 40% when treated and 100% when untreated. There is currently scarce literature available depicting detection and management of pyopericardium. Here we present a case of pyopericardium complicated by cardiac tamponade in an immunocompetent middle-aged man concomitantly diagnosed with community-acquired pneumonia. We also provide a review of current guidelines and literature as they pertain to diagnosis and management of this rare entity.

### CASE PRESENTATION

A 58-year-old male with a past medical history of recent outpatient-treated community-acquired pneumonia was admitted for dyspnea and pleuritic chest pain. Chest x-ray revealed a left-sided pleural effusion and right lower lobe pneumonia. Initial electrocardiogram (ECG) revealed ST-segment elevations in the anterolateral leads with diffuse PR depressions. The patient underwent left heart catheterization, which revealed no obstructive lesions. Ceftriaxone and doxycycline were started in light of persistent pneumonia. Several hours later, the patient’s dyspnea worsened. Bedside echocardiogram demonstrated a moderate free-flowing pericardial effusion with tamponade. A subxiphoid pericardial window and left-sided thoracostomy tube were placed, during which 400cc of purulent material was drained. His antibiotic regimen was escalated to vancomycin, ceftriaxone, and linezolid. Pericardial fluid cultures resulted positive for pan-sensitive Streptococcus pneumoniae, and antibiotic therapy was changed to ceftriaxone. Repeat echocardiogram showed complete resolution of the pericardial effusion. The patient was ultimately discharged to a long-term acute care facility with a prolonged course of antibiotics.

### DISCUSSION

Purulent pericarditis is rarely seen due to early initiation of antibiotics and emphasis on source control. Despite receiving appropriate antibiotics on admission, our patient’s disease progressed to this deadly complication. Cases of pyopericardium involving patients with no discernable risk factors remain rare. The most common bacterial pathogen is Staphylococcus aureus, followed by other Gram-positive organisms. Timely diagnosis remains crucial, with echocardiogram remaining the gold standard. When pyopericardium is suspected, urgent pericardial drainage and broad-spectrum antibiotics are indicated. This case highlights the rapid progression of pyopericardium and its complications.

